# Late-life depression symptom dimensions and cognitive functioning in the Longitudinal Aging Study Amsterdam (LASA)

**DOI:** 10.1016/j.jad.2016.05.027

**Published:** 2016-09-01

**Authors:** Anamaria Brailean, Hannie C. Comijs, Marja J. Aartsen, Martin Prince, A. Matthew Prina, Aartjan Beekman, Martijn Huisman

**Affiliations:** a King's College London, Institute of Psychiatry, Psychology and Neuroscience, Department of Health Service and Population Research, Centre for Global Mental Health, London, UK; b VU University Medical Centre, Department of Psychiatry and the EMGO Institute for Health and Care Research, Amsterdam, the Netherlands; c NOVA - Norwegian Social Research, Center for Welfare and Labor Research, Oslo, Norway; d VU University, Department of Sociology, Amsterdam, the Netherlands; e VU University Medical Center, Department of Epidemiology and Biostatistics and the EMGO Institute for Health and Care Research, Department of Sociology, Amsterdam, the Netherlands

**Keywords:** CES-D, Center for Epidemiologic Studies Depression Scale, CFA, Confirmatory Factor Analysis, CFI, Comparative Fit Index, DIF, Differential Item Functioning, DSM, Diagnostic and Statistical Manual of Mental Disorders, GDS, Geriatric Depression Scale, LASA, Longitudinal Aging Study Amsterdam, MDD, Major Depressive Disorder, MIMIC, Multiple Indicators Multiple Causes, RCPM, Raven Colored Progressive Matrices, RMSEA, Root Mean Square Error of Approximation, TLI, Tucker Lewis Index, WLSMV, Weighted Least Squares Mean and Variance-Adjusted, Late-life depression, Depression symptom dimensions, Cognitive aging, Cognitive abilities, Differential item functioning

## Abstract

**Background:**

Depression often co-occurs in late-life in the context of declining cognitive functions, but it is not clear whether specific depression symptom dimensions are differentially associated with cognitive abilities.

**Methods:**

The study sample comprised 3107 community-dwelling older adults from the Longitudinal Aging Study Amsterdam (LASA). We applied a Multiple Indicators Multiple Causes (MIMIC) model to examine the association between cognitive abilities and latent dimensions of the Center for Epidemiologic Studies Depression Scale (CES-D), while accounting for differential item functioning (DIF) due to age, gender and cognitive function levels.

**Results:**

A factor structure consisting of somatic symptoms, positive affect, depressed affect, and interpersonal difficulties fitted the data well. Higher levels of inductive reasoning were significantly associated with lower levels of depressed affect and somatic symptoms, whereas faster processing speed was significantly associated with lower levels of somatic symptoms. DIF due to age and gender was found, but the magnitude of the effects was small and did not alter substantive conclusions.

**Limitations:**

Due to the cross-sectional context of this investigation, the direction of influence between depression symptom levels and cognitive function levels cannot be established. Furthermore, findings are relevant to non-clinical populations, and they do not clarify whether certain DIF effects may be found only at high or low levels of depression.

**Conclusions:**

Our findings suggest differential associations between late-life depression dimensions and cognitive abilities in old age, and point towards potential etiological mechanisms that may underline these associations. These findings carry implications for the prognosis of cognitive outcomes in depressed older adults.

## Highlights

• Better inductive reasoning was associated with lower severity of depressed affect.
• Better inductive reasoning was associated with lower severity of somatic symptoms.
• Faster processing speed was associated with lower severity of somatic symptoms.
• DIF due to age and gender was statistically significant but of small magnitude.
• No DIF due to level of cognitive functioning was found.

## 1 Introduction

According to the Diagnostic and Statistical Manual of Mental Disorders, Fourth Edition, Text Revision, depression is a multi-dimensional construct consisting of depressed affect (i.e., dysphoria), low positive affect (i.e., anhedonia), and somatic symptoms (American Psychiatric Association, 2000). Compared to their younger counterparts, older adults have a lower prevalence of Major Depressive Disorder (Kessler et al., 2010), but a higher prevalence of subsyndromal depression (Meeks et al., 2011). Also, older adults express lower levels of depressed affect (e.g., feeling sad), but more pronounced somatic symptoms (e.g., fatigue, sleep disturbance, loss of appetite) and motivational symptoms (e.g., lack of interest or enjoyment) (Gallo et al., 1997, Hegeman et al., 2012a, Hegeman et al., 2012b). Studies conducted in older adults suggest a differential association between specific late-life depression dimensions (e.g., depressed affect, positive affect, somatic and motivational symptoms) and various health outcomes, such as brain function (Kirton et al., 2014), functional disability and distress (Gallo et al., 1997), mortality (Blazer and Hybels, 2004, Gallo et al., 1997, John and Montgomery, 2009), cognitive impairment and decline (e.g., Baune et al., 2007, Castro-Costa et al., 2007, Turner et al., 2015), and incident dementia (Lugtenburg et al., 2016). A dimensional approach of depression could help clarify how different symptom presentations relate to various aging-related health outcomes and what etiological mechanisms may underlie these associations.

Although extensive evidence suggests that late-life depression co-occurs with impairment and decline in cognitive abilities such as memory, executive function, processing speed, and visuo-spatial abilities (e.g., Baudic et al., 2004, Comijs et al., 2001, Lockwood et al., 2002, Sheline et al., 2006), it is not clear whether the nature and severity of cognitive impairment differs among persons with specific symptom presentations. Existing reports suggest that motivational and somatic symptoms may be more strongly associated with vascular and degenerative processes (Naarding et al., 2005), as well as with cognitive impairment and Alzheimer Disease (Bartolini et al., 2005, Berger et al., 1999, Gallo et al., 1997, Kumar et al., 2006, Potvin et al., 2010), compared to mood symptoms. Ageing-related dysfunctions in fronto-striatal structures and cerebrovascular disease have been postulated as possible mechanisms underlying the co-occurrence of executive dysfunction and motivational symptoms of depression (Alexopoulos, 2001). Instead, dysphoric symptoms may manifest as a psychological reaction to perceived cognitive decline in the early stages of impairment when older adults are aware of their cognitive dysfunctions. Low positive affect could also affect cognitive functioning by influencing dopamine levels (Ashby et al., 1999), cardiovascular risk (Davidson et al., 2010), and the attention scope (Fredrickson, 2001). The pattern of associations between specific depression dimensions and cognitive functioning and the neuro-biological and psychological mechanisms that may underlie these associations are poorly understood. This is partly due to the scarcity of studies that employed a dimensional approach to depression, and partly due to methodological differences such as the assessment of depression symptom dimensions based on factor analytic studies of different depression scales, and the inclusion of clinically depressed patients or community-dwelling older adults.

Cross-sectional studies conducted in non-clinical populations using the Center for Epidemiologic Studies Depression Scale (CES-D) (Radloff, 1977) suggest that: lower levels of depressed affect and somatic symptoms were related to better performance on tasks assessing speed, attention and executive function, whereas higher levels of positive affect were related to poorer verbal fluency performance (Baune et al., 2007); higher levels of positive affect (but not lower levels of depressed affect, somatic symptoms or interpersonal difficulties) were related to better everyday problem solving (Paterson et al., 2015); positive affect was the most robust predictor of cognitive performance across a variety of tasks assessing memory, processing speed, verbal fluency, visual retention, temporal orientation, and global cognition (La Rue et al., 1995). Studies using the Euro-D depression scale (Prince et al., 1999) found that verbal fluency performance was more strongly associated with motivational symptoms of depression than with affective suffering symptoms (Brailean et al.; Castro-Costa et al., 2007). Cross-sectional studies conducted in persons with MDD suggest that greater levels of apathy on the Hamilton Psychiatric Rating Scale for Depression (Williams, 1988) were associated with lower executive function and processing speed performance (Feil et al., 2003); greater levels of motivational symptoms on the Inventory of Depressive Symptomatology (Rush et al., 1996) were associated with poorer episodic memory and processing speed, whereas greater levels of mood symptoms were associated with poorer working memory and processing speed (Korten et al., 2014). A longitudinal study by Turner et al. (2015) suggests that lower levels of positive affect on the CES-D scale predicted decline in global cognition, episodic memory, and perceptual speed, whereas higher levels of anhedonic symptoms on the Geriatric Depression Scale (GDS) (Yesavage et al., 1982) predicted steeper decline in episodic memory, and higher levels of negative affect on the GDS predicted steeper decline in global cognition, as well as in episodic, semantic, and working memory.

Cognitive function levels may influence not only the severity of depressive symptoms, but also the type of symptoms reported. Given similar levels of depression severity, persons with poor cognitive functioning may be more likely to endorse certain items (e.g., concentration difficulties) than persons with normal cognitive functioning. Previous studies suggest that cognitive status may be related to response bias to several items assessing depression (Fieo et al., 2015, Mast, 2005). If items from depression scales measure different constructs in persons with low versus high cognitive functioning, measurement bias can impact on conclusions about the association between late-life depression and cognitive ageing; hence, the influence of measurement bias should be accounted for. In light of previous findings, it is also important to account for differences in response behavior due to age and gender. For instance, there is evidence that older persons tend to under-report dysphoria and sadness (Gallo et al., 1994, Gallo et al., 1999), and to over-report sleep difficulties, hopelessness, loss of interest, and slowing down (Christensen et al., 1999), whereas women are more likely than men to report having crying spells (Carleton et al., 2013, Yang and Jones, 2007, Yang et al., 2009), and less likely to report feeling like a failure (Yang et al., 2009).

The main aim of this study is to examine whether CES-D depression symptom dimensions (i.e., depressed affect, positive affect, somatic symptoms, and interpersonal difficulties) are differentially associated with performance in specific cognitive domains which are typically altered in late life depression (i.e., inductive reasoning, processing speed, immediate recall and delayed recall). A related aim is to examine item response biases due to age, gender and levels of cognitive functioning, and the extent to which item response biases affect the association between depression symptom dimensions and cognitive abilities.

## 2 Methods

### 2.1 Participants

Data were used from the Longitudinal Ageing Study Amsterdam **(**LASA) (Huisman et al., 2011), an ongoing study exploring physical, emotional, cognitive and social functioning in late life. Respondents were recruited from the population registers of 11 municipalities from three regions in the Netherlands and were interviewed in their homes by trained persons. The LASA study was approved by the Ethical Review Board of the VU University Medical Center and all respondents provided informed consent. The current study used data collected in 1992–1993 (LASA cycle wave B) from respondents aged 55–85 years old (N=3107). This data cycle (i.e., baseline assessment for the first LASA cohort) was selected because it included a larger sample size and a smaller amount of missing data.

### 2.2 Instruments

**Depressive symptoms** were measured using the CES-D (Radloff, 1977). Symptoms are assessed over the course of the past week and ratings to each item are based on a four-point scale 0-3). The total score of the 20 items ranges from 0 to 60, higher scores indicating more depressive symptoms. CES-D has good psychometric properties in older adults (Hertzog et al., 1990, Himmelfarb and Murrell, 1983, McCallum et al., 1995). Good criterion validity was found when using a cut-off score of 16 to identify persons with major depression in LASA (Beekman et al., 1997). The scale consists of four factors: depressed affect, positive affect, somatic symptoms and interpersonal difficulties (Beekman et al., 1994, Radloff, 1977). Higher values on the depressed affect, somatic symptoms and interpersonal difficulties subscales indicate a greater severity of depressive symptoms, whereas higher scores on the positive affect subscale indicate higher levels of positive affect. Previous studies either provided support for the validity of the 4-factor model (for a meta-analysis see Shafer, 2006), or called into question its validity due to a few items displaying bias or not being in line with the current diagnosis criteria for depression (for a review see Carleton et al., 2013).

The present study included all cognitive tests available in LASA which assessed specific cognitive abilities rather than general cognitive performance.

**Episodic memory** was assessed using the 15 Words Test, a Dutch version of the Auditory Verbal Learning Test (Rey, 1964). Participants were verbally presented with 15 words which were repeated over 3 trials. After each trial participants were asked to repeat the words they remembered. Immediate recall performance was determined based on the total score on the three trials. After a distraction period of about 20 min, during which a non-verbal task was performed, participants were asked to name again the words they remembered. This was used as a measure of delayed recall.

**Information processing speed** was assessed in LASA using an adaptation of the Coding Task (Savage, 1984). Participants were shown two rows of characters, each character in the bottom row belonging to a character in the upper row. This correct letter combination was presented at the top of the page together with two other rows, the upper one containing characters and the lower one being empty. Participants were asked to name the character in the bottom row which belonged to the character in the upper row. They were instructed to respond to the letter combinations as quickly and accurately as possible. The test consisted of three trials of 1 min each and the score on each trial was calculated based on the number of completed combinations. The total score for the three trials was used. The coding task is primarily a measure of information processing speed, but also a global measure of intellectual functioning, as the execution of this task involves various cognitive abilities (i.e., attention, memory function, perceptual organization and speed) (Bouma et al., 1996). Because the original task was adapted to require verbal rather than motor responses, it is considered that the test measures cognitive speed rather than motor speed processes.

**Inductive reasoning** was assessed using the Raven Colored Progressive Matrices (RCPM) (Raven, 1995). Performance on the RCPM task requires non-verbal and abstract reasoning which are components of fluid intelligence or executive functioning. On each trial participants are presented with a drawing from which a section is missing. They have to identify the correct missing section from six alternatives patters presented at the bottom of the page. Raven consists originally of three subsets: A, Ab and B. Each subset consists of 12 items and a correct response to each item counts for one point. Both items and subsets show a progressive increase in difficulty. Due to time restrictions, only subsets A and B were used in LASA. The omission of the Ab subset is unlikely to affect test performance as pilot studies in LASA have shown that the sum score of A and B subsets correlates strongly (*r*=0.96) with the sum score of A, Ab and B subsets. The sum score of A and B subsets ranged from 0 to 24. Poor performance on this task is considered a good marker of dementia (Gainotti et al., 1992).

Based on previous research (Alexopoulos, 2005, Blazer, 2003, van den Kommer et al., 2013), the following covariates were considered as potential confounders of the association between cognitive abilities and depression symptom dimensions: age (in years), gender, education (in years), number of chronic diseases (based on self reports of the following disorders: chronic non-specific lung disease, cardiac disease, peripheral arterial disease, diabetes mellitus, cerebrovascular accident or stroke, osteoarthritis, rheumatoid arthritis, cancer, and maximum 2 other disorders), alcohol use (no, middle, and high consumption according to the Netherlands Economic Institute index), exercise (total time spent on physical activities in minutes per day), partner status (having a partner or not), use of antidepressant and anxiolytic medication (user versus non-user, based on an inspection of medicine bottles during the medical interview).

### 2.3 Statistical analysis

**Fig. 1:**
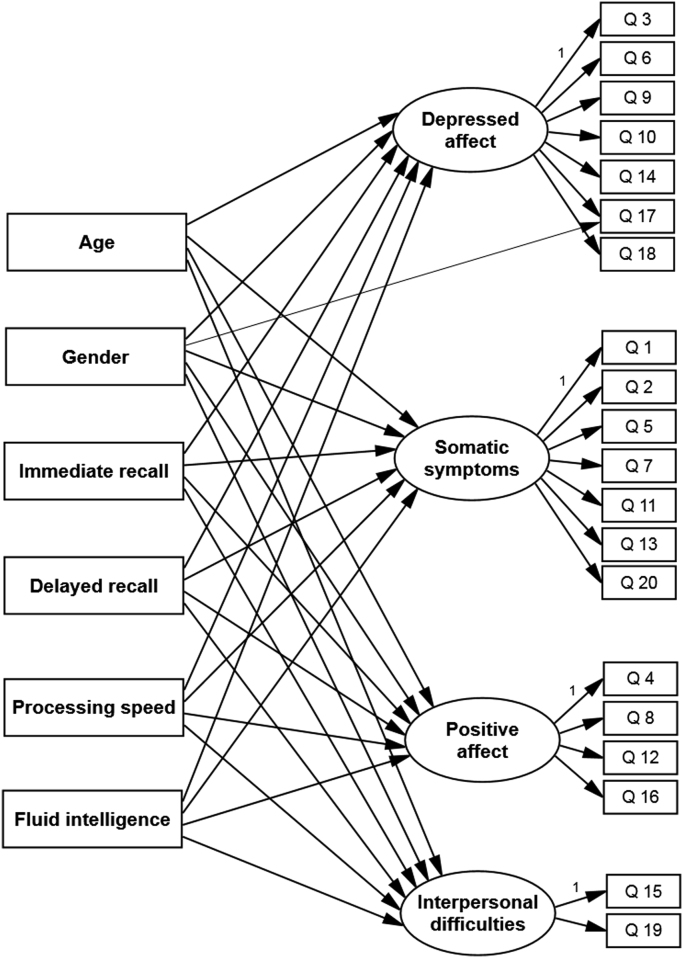
MIMIC model showing the impact of age, gender and cognitive abilities on the CES-D measurement model with four factors. Due to space constraints, residual variances are not presented in the figure. Also, only one example of direct effect is illustrated, indicating gender differences in the probability of endorsing the CES-D item 17 (crying spells).

All analyses were conducted in MPlus Version 7.1 (Muthén and Muthén, 1998–2012). We used mean and variance-adjusted weighted least squares (WLSMV) estimation which can deal with missing data under the assumption that data are missing at random with respect to covariates included in the model (also referred to as MARX assumption). Cases with both complete and partially missing data were used to calculate the correlation matrix. WLSMV estimation can be useful in dealing with missing data under the MARX assumption when the percentage of missing data is not substantial (Asparouhov and Muthen, 2014). First, we conducted confirmatory factor analysis (CFA) to examine the fit of a measurement model with four dimensions: depressed affect, positive affect, somatic symptoms, interpersonal difficulties (Beekman et al., 1994, Radloff, 1977). Model fit was evaluated based on the model Chi-square with a *p* value above 0.05 indicating good model fit (Hu and Bentler, 1999); the comparative fit index (CFI) (Bentler, 1990) and the Tucker Lewis index (TLI) (Tucker and Lewis, 1973) with values above 0.90 suggesting acceptable fit, and values above 0.95 indicating a good fit; the root mean square error of approximation (RMSEA) (Steiger, 1990) with values under 0.06 indicating good fit. After establishing the CFA model, we conducted Multiple Indicators Multiple Causes (MIMIC) modelling (Muthén and Muthén, 1998–2012) which simultaneously estimates: a measurement model specifying the relation between CES-D items and latent depression constructs (i.e., CFA model); a regression model whereby latent depression constructs are regressed on several covariates (i.e., age, gender, cognitive abilities); “direct effects” between CES-D items and covariates which inform on differences in item responses due to group membership, despite similar levels of depression severity between groups (see Figure 1). The presence of direct effects indicates measurement non-invariance or differential item functioning (DIF). For instance, in the context of adjustment for gender differences in the severity of depressed affect, males have a lower probability of responding “Yes” to the item “Have you cried at all?”.

Our initial MIMIC model consisted of the CFA measurement model previously established and a regression model estimating the simultaneous effect of age, gender, immediate recall, delayed recall, inductive reasoning and processing speed on CES-D factor means. Because scores on different cognitive measures are positively correlated in the population we took into account their shared variance by simultaneously estimating the effect of all cognitive abilities on CES-D factors. The initial MIMIC model presumed no DIF in any CES-D item and it served as a baseline model for an inspection of modification indices which informed on how much improvement we would gain in model fit by estimating certain DIF effects due to age, gender, or level of performance in the cognitive abilities assessed. This model also informed about the robustness of the CES-D factor structure in the presence of covariates, and about any differences in CES-D factor means due to age, gender and cognitive function levels.

In a second stage, DIF effects due to age, gender and cognitive function levels were progressively added to the model, starting with the effect leading to the largest improvement in model fit. Model comparison was conducted using a DIFFTEST approach (Muthén and Muthén, 1998–2012) in order to determine whether the adjustment for each additional direct effect resulted in a significant improvement in model fit (i.e., a drop in model Chi square values). Given that the modelling framework involved WLSMV estimation, probit regression coefficients were obtained for direct effects. After adding all significant direct effects to the model, we examined the impact of this adjustment on the association of CES-D factor means with cognitive abilities, age, and gender.

In a third stage, we re-examined the association between CES-D factor means and cognitive abilities (including all direct effects) after adjustment for the effect of eight potential confounders of the association between depression dimensions and cognitive domains: education, number of chronic diseases, alcohol use, exercise, smoking, partner status, use of antidepressant, and use of anxiolytic medication. First, all these covariates were included as predictors in the MIMIC model (alongside with age, gender and cognitive abilities). An additional set of models estimated the effect of one covariate at a time (alongside with the effect of age, gender and cognitive abilities) on depression dimensions in order to determine which covariates could account for the observed associations between cognitive abilities and CES-D depression-dimensions.

Because cognitive and depression measures may lack reliability and validity in persons with cognitive impairment or dementia, we conducted sensitivity analyses in a subsample that excluded participants with potential cognitive impairment (i.e., a score of 23 and below on the MMSE).

## 3 Results

Descriptive statistics for our sample are presented in Table 1. Because our study included data from the baseline LASA cycle, the percentage of missing data was low. Less than 5% of participants had missing data on any one CES-D item, and less than 2% of participants had missing data on the total CES-D score. Sixteen percent of participants had clinically significant depressive symptoms (i.e., a CES-D score of 16 and above). Eleven percent of participants had at least mild to moderate cognitive impairment (i.e., a score of 23 and below on the Mini Mental State Examination).

Confirmatory Factor Analysis was conducted to test a model with 4 factors: somatic symptoms, positive affect, depressed affect, and interpersonal difficulties. CFA results are presented in Table 2. The measurement model showed good fit: Chi square=1469.53, df 165, p<0.001; CFI=0.96; TLI=0.95; RMSEA =0.05 (95% confidence interval=0.05–0.05), and all CES-D items loaded well on the hypothesized factors. The depressed affect factor had a correlation of *r*=−0.75 with positive affect, *r*=0.86 with somatic symptoms, and *r*=0.49 with interpersonal difficulties. Positive affect had a correlation of *r*=−0.69 with somatic symptoms, and *r*=−0.33 with interpersonal difficulties. Somatic symptoms had a correlation of *r*=0.52 with interpersonal difficulties. Given that the hypothesized factor structure fitted the data well, this measurement model was included in our MIMIC models.

Our initial MIMIC model results showed that the CFA model for CES-D is robust to external covariates (i.e., cognitive abilities, age and gender). The model fitted the data well: CFI=0.95; TLI=0.94; RMSEA=0.04 (95% confidence interval=0.04 to 0.04), and factor loadings remained strong and statistically significant (results not presented). Model results suggest that persons with lower levels of inductive reasoning had statistically significant higher levels of depressed affect and somatic symptoms, whereas persons with slower processing speed had statistically significant higher levels of somatic symptoms (see model 1 in Table 4). A marginally significant association was found between processing speed and depressed affect (B<−0.01, p=0.06, β=−0.06), suggesting that slower processing speed was related to higher levels of depressed affect. Females had statistically significant higher levels of depressed affect (B=0.42, p<0.001, β=0.47) and somatic symptoms (B=0.30, p<0.001, β=0.44), as well as lower levels of positive affect (B=−0.19, p<0.001, β=−0.30). Older persons had statistically significant lower levels of positive affect (B=−0.01, p<0.01, β=−0.09).

In the context of adjustment for the severity of depression symptom dimensions, three items displayed response bias due to gender, indicating that women had a higher probability of reporting crying spells (item 17), sleep disturbance (item 11), and feeling as good as others (item 4) (see Table 3). Two items displayed response bias due to age, indicating that older persons reported more loneliness (item 14) and less hope about the future (item 8) (see Table 3). We found no DIF due to level of cognitive functioning. DIFFTEST results indicated a significant drop in model Chi square for each additional DIF effect estimated (suggesting an improvement in model fit). Adjusting for DIF effects did not alter conclusions about the associations of CES-D factors with cognitive abilities (see model 2 in Table 4). Lower levels of inductive reasoning remained statistically significant associated with higher levels of depressed affect and somatic symptoms, whereas slower processing speed remained statistically significant associated with higher levels of somatic symptoms. Also, age and gender differences in CES-D factor means remained significant and of similar magnitude after adjusting for DIF (results not presented).

After adjusting for the effects of additional covariates (i.e., education, number of chronic diseases, alcohol use, exercise, smoking, partner status, use of antidepressant and anxiolytic medication), the association between processing speed and somatic symptoms was no longer statistically significant, whereas persons with lower levels of inductive reasoning continued to show higher levels of depressed affect and somatic symptoms (see model 3 in Table 4). Additional analyses were conducted to determine which of the covariates accounted for the observed associations between depression dimensions and cognitive domains. The association between somatic symptoms and processing speed lost statistical significant after accounting for the number of chronic diseases, but it remained significant or marginally significant when adjusting for any of the other covariates. Inductive reasoning remained statistically significant associated with depressed affect and somatic symptoms after adjusting for any of the confounders. Findings from sensitivity analyses conducted in older adults without potential cognitive impairment suggest that the CES-D factor structure, the DIF effects, and the associations between depression-symptom dimensions and cognitive abilities did not change as a result of excluding cognitively impaired persons.

## 4 Discussion

Using data from a large nationally representative sample of LASA and an analytic strategy that adjusted for the influence of measurement bias, our findings provide partial support for a differential association between depression symptom dimensions and cognitive abilities, while also adding evidence for the measurement invariance of CES-D across age, gender and cognitive function levels in older adults. Consistent with previous studies (Radloff, 1977, Shafer, 2006), we found that depression, as measured by CES-D, can be interpreted in terms of four domains: depressed affect, positive affect, somatic symptoms and interpersonal difficulties. Findings from our MIMIC models suggest that depression symptom dimensions are, to some extent, differentially related to cognitive functioning. Higher levels of depressed affect and somatic symptoms were associated with lower levels of inductive reasoning. Higher levels of somatic symptoms were associated with slower processing speed, but this effect was no longer significant after accounting for the number of chronic diseases. Depressed affect was also associated with processing speed but the effect was only marginally significant. Positive affect and interpersonal difficulties were unrelated to any domains of cognitive performance. Although we found differences in response behavior due to age and gender, the magnitude of item response biases was small and did not affect substantive conclusions about the association between depression dimensions and cognitive abilities. These results have some interesting implications which are discussed below.

Our findings that depressed affect and somatic symptoms were similarly associated with inductive reasoning, and that there was a tendency for both symptom dimensions to be associated with processing speed, are consistent with evidence by Baune et al. (2007) suggesting that higher levels of depressed affect and somatic symptoms were associated with poorer performance on tasks assessing speed, attention and executive function. These findings are in agreement with the subcortical-frontal circuit dysfunction model of late-life depression (Butters et al., 2004), and they build upon existing evidence that late-life depression co-occurs with cognitive impairment affecting in particular executive function and processing speed abilities (Lockwood et al., 2002, Sheline et al., 2006). Our finding that processing speed performance was associated with the somatic factor of CES-D (assessing a combination of somatic and motivational symptoms of depression) is consistent with findings from Euro-D studies suggesting a specific association between motivational symptoms of depression and poor processing speed on a verbal fluency task (i.e., naming as many animals as possible in one minute) (Brailean et al., 2015, Castro-Costa et al., 2007). The specific association between somatic/motivational symptoms and slow processing speed may be due to vascular disease and a disruption of frontal-subcortical pathways (Alexopoulos, 2002, Alexopoulos et al., 1997), or it may suggest psychomotor slowing due to co-morbid medical conditions. The finding that the number of chronic diseases accounted for the association between somatic symptoms and processing speed in our study is not surprising given in light of evidence that chronic diseases are associated with slower processing speed (Comijs et al., 2009), and with an increased risk of depression (Huang et al., 2010). It is of note that depressed affect and somatic symptoms were highly correlated, which may explain their co-occurrence with poor cognitive functioning. Albeit statistically significant, all effects were small, which is possibly due to the inclusion of relatively healthy community dwelling older adults in our study (i.e., only a small percentage of participants met criteria for possible cognitive impairment or clinical depression).

Because scores on different cognitive measures are positively correlated in the population, our models controlled for their mutual variance in order to determine the unique effect of each cognitive ability on depression scores. The finding that recall ability was not associated with scores on any of the depression factors after partialling out the effect of inductive reasoning and processing speed is consistent with evidence that age differences in memory performance are reduced or eliminated when controlling for processing speed (Bunce and Macready, 2005) or for executive function (Clarys et al., 2009). The finding that the interpersonal difficulties and positive affect dimensions were unrelated to any domains of cognitive performance may suggest that these symptoms of depression are less strongly related to brain changes underlying aging-related cognitive decline, or that these dimensions of CES-D may capture constructs that are not core symptoms of depression.

With regard to differential item functioning, our findings suggest that levels of cognitive functioning did not influence the probability of endorsing certain CES-D items. This could be explained by our sample composition in which only 11% of participants showed cognitive impairment. In the absence of genuine differences in the severity of depression dimensions, older persons reported more loneliness and less hope about the future than younger persons. Females were more likely to report feeling as good as others, sleep disturbance, and crying spells. Our findings are consistent with previous studies reporting a higher probability of endorsing the item “I had crying spells” in women (Callahan and Wolinsky, 1994, Carleton et al., 2013, Yang and Jones, 2007, Yang et al., 2009), and with studies showing higher levels of hopelessness (Christensen et al., 1999), as well as increased loneliness at advanced ages (Dykstra, 2009). The finding that the magnitude of all DIF effects was small and adjusting for these effects did not alter our substantive conclusions suggests that CES-D has similar measurement properties and can be usefully employed across age, gender and cognitive function levels.

A notable strength of this study is the use of an analytic strategy involving latent variables modelling which allows to account for measurement error and to test for DIF effects while simultaneously investigating the effect of multiple predictors (measured continuously or categorically) on CES-D depression dimensions (Woods, 2009). Other study strengths include the large sample size which allowed for adjustment for important confounders of the association between depression symptoms and cognitive functioning, and the dimensional approach to late-life depression. Our study has also a number of limitations. First, the analytic approach employed can only inform on differences in response behavior due to age, gender and cognitive functioning that are constant across levels of depression symptoms (i.e., uniform DIF). Studies using alternative analytic methods (i.e., multi-group models) could examine whether inconsistencies in response behavior occur only at high or at low levels of depression severity (i.e., non-uniform DIF). Second, the cross-sectional context of this investigation cannot inform on the direction of influence between late-life depression dimensions and cognitive functions. Studies employing a longitudinal design are needed to help clarify the extent to which specific symptom dimensions could differentially predict cognitive outcomes in late life. Last but not least, our study assessed cognitive functioning and depressive symptoms on a continuum of severity, with only a small percentage of persons meeting criteria for possible cognitive impairment or clinical depression. Therefore, our conclusions are limited to community dwelling older adults and more research is needed to determine the extent to which our findings can be replicated in clinical samples.

There remains a need for future studies to harmonize depression symptom dimensions derived from factor analytic studies of different depression measures, and to determine the predictive validity of these depression dimensions by examining the extent to which they are differentially related to cognitive outcomes and to other health outcomes in late life. Studies using a bifactor approach (Reise, 2012) could also help disentangle specific dimensions of depression that are independent from a general factor of depression. A better understanding of the association between cognitive functioning and particular symptom dimensions of late-life depression could benefit future research by informing on potential etiological mechanisms underlying the co-morbid manifestation of depression and cognitive impairment, by helping predict cognitive outcomes in patients with specific depression symptom profiles, and by encouraging the development of targeted interventions for depressed older adults.

## Role of the funding source

This work was supported by the Marie Curie Initial Training Network project MARATONE (Grant number: MC ITN-316795 to A. Brailean); The Netherlands Ministry of Health Welfare and Sports, Directorate of Long-Term Care; MRC (MR/K021907/1 to A. M. Prina). The funding sources had no involvement in the conduct of research or in the preparation of the article.

## Contributors

The authors contributed to this study as follows: Anamaria Brailean – literature searches, study design, data analysis and interpretation, writing the manuscript; Dr. Hannie C. Comijs – data management, interpretation of results, revising the manuscript; Dr. Marja J. Aartsen - interpretation of results, revising the manuscript; Prof. Martin Prince – interpretation of results, revising the manuscript; Dr. Matthew Prina – interpretation of results, revising the manuscript; Prof. Aartjan Beekman - interpretation of results, revising the manuscript; Dr. Martijn Huisman - data management, interpretation of results, revising the manuscript. All authors contributed to and have approved the final manuscript.

## Conflict of interest

none.

## Figures and Tables

**Table 1.** Characteristics of the study sample.

**Continuous measures**	**N**	**Mean**	**S.D.**	**Categorical measures**		**N**	**%**
Age	3107	70.8	8.8	Sex	Female	1601	52
Education	3099	8.8	3.3		Male	1506	48
Chronic diseases	3087	1.4	1.2	Partner status	With partner	2061	66
Physical activity	2889	54.0	69.3		Single	1043	34
Depressive symptoms	3056	7.97	7.79	Antidepressants	User	51	2
Immediate recall	2615	18.4	6.3		No user	2620	98
Delayed recall	2615	5.0	2.8	Anxiolytics	User	2507	94
Inductive reasoning	2821	17.7	4.1		No user	164	6
Processing speed	2565	71.7	22.7	Smoking	Never smoked	823	31
General cognitive ability	3091	26.8	3.2		Past smoker	1169	44
					Current smoker	672	25
				Alcohol use	None	592	22
					Moderate	1842	69
					Severe	220	8

*Note:* Age and education were measured in years; physical activity was measured in minutes per day; total scores were used for number of chronic diseases and for cognitive measures; immediate and delayed recall - Auditory Verbal Learning Test; inductive reasoning - Raven Colored Progressive Matrices; processing speed – Coding Task; general cognitive performance – Mini Mental State Examination; depressive symptoms - Center for Epidemiologic Studies Depression Scale.

**Table 2.** CFA model results for CES-D items.

**Item no.**	**Item content**	**β**	**S.E.**
		**Depressed affect**	
CESD 3	I could not shake off the blues	0.85	0.01
CESD 6	I felt depressed	0.88	0.01
CESD 9	I felt my life was a failure	0.70	0.03
CESD 10	I felt fearful	0.71	0.02
CESD 14	I felt lonely	0.77	0.02
CESD 17	I had crying spells	0.75	0.02
CESD 18	I felt sad	0.84	0.01
		**Positive affect**	
CESD 04	I felt that I was just as good as other people	0.60	0.02
CESD 08	I felt hopeful about the future	0.54	0.02
CESD 12	I was happy	0.85	0.01
CESD 16	I enjoyed life	0.91	0.01
		**Somatic symptoms**	
CESD 01	I was bothered by things that usually don't bother me	0.65	0.02
CESD 02	My appetite was poor	0.57	0.03
CESD 05	I had trouble keeping my mind on what I was doing	0.64	0.02
CESD 07	I felt that everything I did was an effort	0.78	0.01
CESD 11	My sleep was restless	0.56	0.02
CESD 13	I talked less than usual	0.58	0.02
CESD 20	I could not get "going"	0.67	0.02
		**Interpersonal difficulties**	
CESD 15	People were unfriendly	1.00	<0.01
CESD 19	I felt that people dislike me	0.84	0.03

*Note:* β=standardized coefficients; All factor loadings are significant at p<0.001.

**Table 4.** Cross-sectional associations between CES-D factors and cognitive abilities.

	**Depressed affect**			**Positive affect**			**Somatic symptoms**			**Interpersonal difficulties**		
	**B**	**S.E.**	**β**	**B**	**S.E.**	**β**	**B**	**S.E.**	**β**	**B**	**S.E.**	**β**
**Model 1. Effect of cognitive abilities on CES-D factors in a model unadjusted for DIF**												
Immediate recall	−<0.01	0.01	−0.03	0.01	<0.01	0.04	<−0.01	0.01	−0.01	−0.01	0.01	−0.05
Delayed recall	0.01	0.01	0.04	<−0.01	0.01	<−0.01	<0.01	0.01	0.02	−0.01	0.02	−0.02
Inductive reasoning	−0.03***	0.01	−0.11	<0.01	0.01	0.01	−0.02***	0.01	−0.12	−0.02	0.01	−0.07
Processing speed	<−0.01	<0.01	−0.06	<0.01	<0.01	0.03	<−0.01*	<0.01	−0.07	<0.01	<0.01	0.04
**Model 2. Effect of cognitive abilities on CES-D factors in a model adjusted for DIF**												
Immediate recall	−<0.01	0.01	−0.03	0.01	<0.01	0.04	<−0.01	0.01	−0.01	−0.01	0.01	−0.05
Delayed recall	0.01	0.01	0.04	<−0.01	0.01	−0.01	<0.01	0.01	0.02	−0.01	0.02	−0.02
Inductive reasoning	−0.03***	0.01	−0.11	<0.01	0.01	0.01	−0.02***	0.01	−0.12	−0.02	0.01	−0.07
Processing speed	<−0.01	<0.01	−0.06	<0.01	<0.01	0.03	<−0.01*	<0.01	−0.07	<0.01	<0.01	0.04
**Model 3. Effect of cognitive abilities on CES-D factors in models adjusted for DIF and for the effect of additional covariates**												
Immediate recall	<−0.01	0.01	−0.02	<0.01	<0.01	0.04	<0.01	0.01	<0.01	−0.01	0.01	−0.08
Delayed recall	0.02	0.01	0.05	<−0.01	0.01	−0.01	<0.01	0.01	<−0.01	<0.01	0.02	0.01
Inductive reasoning	−0.02**	0.01	−0.10	<0.01	0.01	<−0.01	−0.02***	0.01	−0.11	−0.02	0.01	−0.07
Processing speed	<−0.01	<0.01	−0.02	<0.01	<0.01	0.02	<−0.01	<0.01	-0.03	<0.01	<0.01	0.02

*Note:*

B=non-standardized coefficients; S.E.=standard error; β=standardized coefficients; for gender the reference group is male; all three models are adjusted for age and gender; additionally, the third model is adjusted for education, number of chronic diseases, alcohol use, smoking, exercise, partner status, use of antidepressant and anxiolytic medication.

* p<0.05;

** p<0.01;

*** p<0.001.

**Table 3.** MIMIC models with direct effects between covariates and CES-D items.

**Number of direct effects**		**chi**^**2**^**(df)**	**Δ chi**^**2**^	**B**	**S.E.**	**β**
	No direct effects	1347 (261)				
1	Age predicts Loneliness (CESD 14)	1313 (260)	41	0.02	<0.01	0.18
2	Gender predicts Crying (CESD 17)	1293 (259)	23	0.29	0.06	0.29
3	Age predicts Hope (CESD 8)	1278 (258)	18	−0.01	<0.01	−0.10
4	Gender predicts Sleep (CESD 11)	1265 (257)	15	0.18	0.05	0.18
5	Gender predicts Feeling as good as others (CESD 4)	1252 (256)	14	0.21	0.06	0.21

*Note:* All direct effects are significant at p<0.001; chi^2^ (df) – model chi squared and the associated degrees of freedom; Δ chi^2^ refers to the difference in chi-square between a model that estimates one additional direct effect and a model that estimates one fewer direct effect; B=non-standardized coefficients; S.E.=standard error; β=standardized coefficients
